# Surveillance for Adenoviruses in Bats in Italy

**DOI:** 10.3390/v11060523

**Published:** 2019-06-06

**Authors:** Georgia Diakoudi, Gianvito Lanave, Ana Moreno, Chiara Chiapponi, Enrica Sozzi, Alice Prosperi, Vittorio Larocca, Michele Losurdo, Nicola Decaro, Vito Martella, Antonio Lavazza, Davide Lelli

**Affiliations:** 1 Dipartimento di Medicina Veterinaria, Università Aldo Moro di Bari, S.p per Casamassima km 3, 70010 Valenzano, Italy; georgiadiakoudi@gmail.com (G.D.); gianvito.lanave@uniba.it (G.L.); laroccavittorio@yahoo.it (V.L.); michele.losurdo@libero.it (M.L.); nicola.decaro@uniba.it (N.D.); vito.martella@uniba.it (V.M.); 2 Istituto Zooprofilattico Sperimentale della Lombardia e dell’Emilia Romagna, Via Bianchi 9, 25124 Brescia, Italy; anamaria.morenomartin@izsler.it (A.M.); chiara.chiapponi@izsler.it (C.C.); enrica.sozzi@izsler.it (E.S.); alice.prosperi@izsler.it (A.P.); antonio.lavazza@izsler.it (A.L.)

**Keywords:** bat, adenovirus, mastadenovirus, aviadenovirus, sequence, NGS, Italy, phylogenetic analysis

## Abstract

Adenoviruses are important pathogens of humans and animals. Bats have been recognized as potential reservoirs of novel viruses, with some viruses being regarded as a possible zoonotic threat to humans. In this study, we report the detection and analysis of adenoviruses from different bat species in northern Italy. Upon sequence and phylogenetic analysis, based on a short diagnostic fragment of the highly-conserved DNA polymerase gene, we identified potential novel candidate adenovirus species, including an avian-like adenovirus strain. An adenovirus isolate was obtained in simian cell lines from the carcass of a *Pipistrellus kuhlii*, and the complete genome sequence was reconstructed using deep sequencing technologies. The virus displayed high nucleotide identity and virtually the same genome organization as the *Pipistrellus pipistrellus* strain PPV1, isolated in Germany in 2007. Gathering data on epidemiology and the genetic diversity of bat adenoviruses may be helpful to better understand their evolution in the mammalian and avian hosts.

## 1. Introduction

Adenoviruses (family *Adenoviridae*) are non-enveloped, icosahedral viruses about 70 to 90 nm in diameter. The linear double stranded DNA genome ranges in length from 26 to 48 kb and contains inverted terminal repeats of 36 to 371 bp [1,2]. The genome of adenoviruses is organized in at least 16 clearly defined genus-common genes, including the polymerase, and in a set of more variable genes, genus-specific, mainly located near the ends the genome [3]. Adenoviruses are classified into five genera by the International Committee on Taxonomy of Viruses (ICTV), namely *Atadenovirus*, *Aviadenovirus*, *Ichtadenovirus*, *Mastadenovirus*, and *Siadenovirus* [4]. The genus, *Mastadenovirus*, includes adenoviruses that infect only mammals, including humans, causing ocular, respiratory, and gastrointestinal diseases [5,6].

Adenoviruses tend to co-evolve with their host [7]. The mechanisms driving their evolution include the accumulation of punctate mutations, homologous recombination, gene capture, and inter-species transmission [3,8]. Evolutionary analysis of adenoviruses of African non-human primates has unveiled that human mastadenovirus B (HAdV-B) circulating in humans are of zoonotic origin, suggesting that multiple independent HAdV-B transmission events to humans occurred more than 100,000 years ago [9].

Bats are potential or confirmed reservoirs of various zoonotic viruses worldwide [10,11]. Several emerging and re-emerging viruses, including lyssaviruses, coronaviruses, henipaviruses, and astroviruses, are harbored by bats and occasionally spread to other mammalian species [12,13,14,15,16]. Due to their marked differences in feeding, habits, and geographical distribution, the various species of bats interact in various ways and with different frequency with other mammals, either directly or indirectly [17,18,19].

Adenoviruses have been discovered only recently in bats. In 2008, a bat adenovirus (BtAdV), strain FBV1 (BtAdV-1) was isolated from a Ryukyu flying fox (*Pteropus dasymallus yayeyamae*) in Japan [20]. Since then, several bat adenoviruses have been identified [21,22,23], including the BtAdV-2 strain PPV1 [24] and BtAdV-3 strain TJM [25]. Attempts to classify BAtdVs have been made, taking into account the apparent genetic heterogeneity, through the bat species of origin and the geographic location. Thus far, all BtAdVs have been included into the genus, *Mastadenovirus*, and seven species have been proposed by ICTV (Table 1).

Adenoviruses have long been considered as host specific viruses that co-evolve with their hosts [3,7]. However, recently, some adenoviruses have been shown to emerge through cross-species transmission, thus highlighting the potential threat of animal-to-animal or animal-to-human circulation of adenoviruses [26,27,28]. In this study, we investigated the circulation of BtAdVs in Italy. A BtAdV isolate was obtained from *Pipistrellus kuhlii* (Kuhl’s pipistrelle) and the full-length genome was sequenced.

## 2. Materials and Methods

### 2.1. Origin of Samples

A total of 195 samples from 8 different bat species (*Pipistrellus kuhlii*, *Pipistrellus pipistrellus*, *Pipistrellus* spp., *Hypsugo savii*, *Plecotus auritus*, *Plecotus* spp., *Tadarida teniotis*, *Eptesicus serotinus*) were collected during 2016 to 2017 in the north of Italy by the Istituto Zooprofilattico Sperimentale della Lombardia ed Emilia Romagna (IZSLER) in the framework of a passive surveillance program aimed to detect viruses in bats. The survey study did not involve any direct manipulations of bats and relied entirely on the collection of carcasses of bats provided by rehabilitation centers. The samples consisted of pools of organs (encephalon, intestine, and viscera) (*n* = 171); a small portion of fecal pellet samples (*n* = 24) were also collected. All samples were stored at −80 °C until use.

### 2.2. DNA Extraction and PCR Amplification

All the samples were homogenized in Minimal Essential Medium (MEM, 1 g/10 mL) containing antibiotics and clarified by centrifugation at 3000 g for 15 min. Viral DNA was extracted from 200 μL of the supernatants using the QIAmp Cador Pathogen Mini Kit (Qiagen S.p.A., Milan, Italy), following the manufacturer’s protocol.

To assess the presence of adenoviral DNA, all samples were screened using a nested-PCR protocol for the amplification of a partial sequence (318–324 bp) of the DNA polymerase gene. For the first amplification, AccuPrime Taq DNA polymerase (Invitrogen, ThermoFisher Scientific, Carlsbad, CA, USA), polFouter forward primer (5’-TNMGNGGNGGNMGNTGYTAYCC-3’), and polRouter reverse primer (5’-GTDGCRAANSHNCCRTABARNGMRTT-3’) were used [29]. Cycling thermal conditions included initial activation of the polymerase at 94 °C for 2 min, 35 cycles at 94 °C for 30 s, 46 °C for 30 s, and 68 °C for 1 min, followed by final extension at 72 °C for 10 min. For the second amplification, 1 μL of product from the first PCR diluted 1:100 diethylpyrocarbonate (DEPC) treated water was used as the nucleic acid template and was amplified under the same conditions using polFinner forward primer (5’-GTNTWYGAYATHTGYGGHATGTAYGC-3’) and polRinner reverse primer (5’-CCANCCBCDRTTRTGNARNGTRA-3’) [29].

PCR products were subjected to electrophoresis on a 1.5% agarose gel containing a fluorescent nucleic acid marker (GelRed; Bio-Rad Laboratories, Hercules, CA, USA) at 80 V for 45 min and visualized under fluorescent light on the Gel Doc EZ Imaging System with Image Laboratory Software (Bio-Rad Laboratories Hercules, CA, USA). The DNA concentration of the samples tested positive was quantified using the Fluorometric Qubit dsDNA High Sensitivity Assay Kit (ThermoFisher Scientific, Waltham, MA, USA). PCR products with satisfying DNA concentrations (>10 ng/μL) were directly sequenced by Eurofins Genomics GmbH (Ebersberg, Germany).

### 2.3. Virus Isolation

For virus isolation, African green monkey kidney derived MARC-145 cells and African green monkey kidney epithelial Vero cells were used. The cells were grown in MEM supplemented with 10% fetal bovine serum (FBS). Pooled tissues from adenovirus-positive samples were homogenized in MEM and then centrifuged at 3000× *g* for 15 min. Supernatants were treated with antibiotics for 30 min (penicillin 5000 IU/mL, streptomycin 2500 μg/mL, amphotericin B 10 μg/mL), inoculated on partially confluent MARC-145 and Vero cell cultures and incubated at 37 °C in a 5% CO_2_ incubator for 7 days to observe the development of the cytopathic effect (CPE). In the absence of CPE, the cryolysate were sub-cultured twice into fresh monolayers. In the presence of the CPE, the cryolysates were examined with negative staining electron microscopy (nsEM) by using the Airfuge (Beckman Instruments, Palo Alto, CA, USA) method [30].

### 2.4. Next-Generation Sequencing

DNA for next-generation sequencing (NGS) was extracted from viral stocks obtained from semi-purified virus particles, in order to sequence the full-length genome of the isolated virus. Briefly, MARC-145 cells were infected with isolate ITA/2018/251170-16. At 48 h post-infection, cell medium was collected and clarified by centrifugation at 1000× g for 10 min at 4 °C. Viral DNA was extracted using the BioSprint 96 One-For-All Vet Kit (Qiagen S.p.A., Milan, Italy) according to the manufacturer’s instructions. A genomic DNA library was prepared using the Nextera DNA Flex Library Prep Kit (Illumina, San Diego, CA, USA) according to the manufacturer’s protocol. Library samples were normalized as suggested by the manufacturer’s instructions and sequencing was performed on the Illumina MiSeq instrument (Illumina, San Diego, CA, USA), using a MiSeq reagent kit v2.

### 2.5. Genome Annotation and Comparison

The total paired reads obtained by the NGS sequencing were checked for quality using FastQC [31]. Sequence trimming, assembly of NGS reads, and genome annotation were performed using Geneious software version 10.2.4 (Biomatters Ltd., Auckland, New Zealand) and the *Bat Mastadenovirus B* strain PPV1 (GenBank accession no. JN252129) as the reference sequence. The full-length genome of the strain ITA/2018/251170-16 was deposited in the GenBank database under the accession number MK625182.

### 2.6. Sequence and Phylogenetic Analysis

Genome sequences of the polymerase coding region of adenovirus and of the full-length adenovirus genome were retrieved from GenBank. The alignment of the sequences was performed using the MAFFT multiple alignment program version 7.388 implemented in the Geneious software (v. 10.2.4). Sequence analysis was conducted using the Geneious v. 10.2.4 software (Biomatters Ltd., Auckland, New Zealand). Phylogenetic analysis was conducted with MEGA-X v. 10.0.5 software [32]. Phylogenetic analysis for the full-length and partial-length adenovirus strains were performed using the maximum likelihood method, Jukes-Cantor genetic distance model, and bootstrapping up to 1000 replicates.

## 3. Results

### 3.1. Molecular Screening

Overall, a total of 34/195 (17.4%) samples tested positive with panadenovirus PCR screening. In detail, from the 195 samples tested, information was available for 142 samples (72.8%) about age, for 130 (66.7%) about sex, and for 195 (100%) about the bat species. AdV DNA was found in 2/62 (3.2%) juvenile bats and 13/80 (16.3%) adult bats. AdV DNA was also found in 19/53 (35.9%) samples of unidentified age. When considering the prevalence based on sex, AdV DNA was detected in 5/54 (9.3%) male and 9/76 (11.8%) female individuals, whilst it was found in 20/65 (30.8%) samples of unidentified sex. Finally, when considering the prevalence of AdV DNA among the various bat species, 11 adenovirus-positive animals were found out of 59 (18.6%) for *Pipistrellus* spp., 8/50 (16.0%) for *Hypsugo savii*, 9/73 (12.3%) for *Pipistrellus kuhlii*, 4/5 (80.0%) for *Eptesicus serotinus*, 1/4 (25.0%) for *Pipistrellus pipistrellus*, 1/1 (100.0%) for *Tadarida teniotis*, 0/2 (0.0%) for *Plecotus auritus*, and 0/1 (0.0%) for *Plecotus* spp.

### 3.2. Virus Isolation

**Figure 1:**
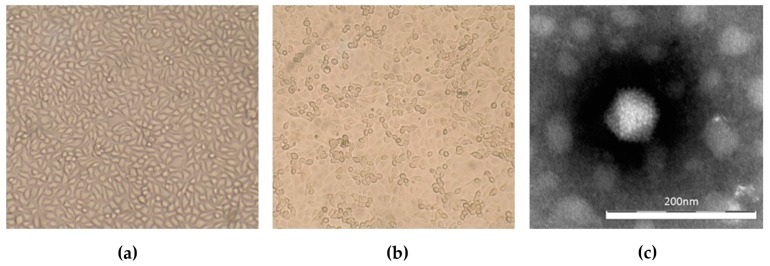
Detection of adenovirus isolate ITA/2018/251170-16 with MARC-145 cells. (**a**) Control cells, 10x magnification; (**b**) Infected cells showing CPE, 10× magnification; (**c**) negative staining (NaPT 2%) electron microscopy of ITA/2018/251170-16 virion. Bar represents 200 nm.

Virus isolation from the adenovirus-positive samples was successful with both MARC-145 and Vero cells only for the sample ITA/2018/251170-16, originating from the pool of organs of a *Pipistrellus kuhlii* found in Lumezzane (BS) with clinical signs of inappetence and sensory depression before death. A clear CPE was observed at the second passage in MARC-145 cells, showing a rounding of cells, increased granularity, and detachment from the monolayer (Figure 1a,b). In Vero cells, the CPE effect was less evident than in MARC-145. The presence of the virus was confirmed in the supernatant of the infected cells at the first passage, using nsEM. Non-enveloped icosahedral virus particles of 80 nm in size were observed that resembled the characteristic morphology of adenoviruses (Figure 1c) [24].

### 3.3. NGS Analysis and Genome Structure of the Adenovirus Strain ITA/2018/251170-16

**Figure 2:**
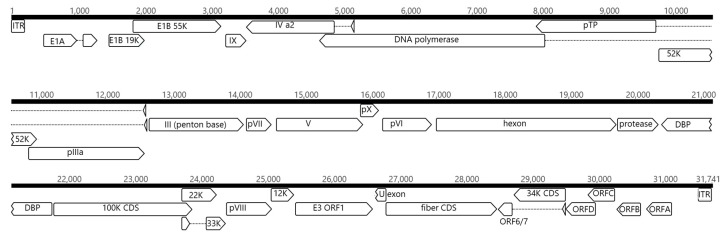
Genomic organization of strain ITA/2018/251170-16. The genome is represented by a black horizontal line marked at 1000-bp intervals. The predicted ORFs are shown as arrows. Dashed lines represent potential splicing mechanisms.

NGS analysis provided the full-length genomic sequence of the adenovirus isolate ITA/2018/251170-16. A total of 82,641 out of 131,270 reads (62.95%), of an average length of 251 bp, were mapped to the reference sequence PPV1 (GenBank accession no. JN252129), with a mean coverage of 518.3 x. A consensus sequence of 31,629 bp was generated, covering the complete genome sequence of the strain PPV1 used as the reference. The sequences were 99.3% identical to each other at the nucleotide level. The full-length sequence of the adenovirus isolate displayed a genome organization similar to that of the reference PPV1 strain, with an average G + C content of 53.5%, an inverted terminal repeat (ITR) of 205 bp, and 31 predicted genes (Figure 2). The three E1 genes (E1A, E1B 19K, and E1B 55K) were present in the Italian isolate (Figure 2). The 16 genus-common core genes, located in the central part of the genome, were conserved [3]. Intronic regions were identified in the IVa2, pTP, DNA polymerase, and 33K genes, as observed in the reference strain PPV1 and in other mastadenoviruses. The E3 region of BtAdV-2, BtAdV-3, and of canine adenoviruses consisted of the gene 12.5K, which is present in the majority of mastadenoviruses, and of the E3 gene, present only in BtAdV-2, BtAdV-3, and in canine adenoviruses.

The U exon, located between the E3 and the fiber genes, was present in the Italian isolate, likewise in many other AdVs [33,34]. In the genome sequence of strain ITA/2018/251170-16, there was a single fiber gene, like in all non-primate mastadenoviruses, in BtAdV-2 and BtAdV-3. The E4 region, adjacent to the fiber gene, is the second most variable region in terms of length and contents in mastadenovirus genomes [34]. This region contains the spliced ORF6/7 gene and a single copy of the highly conserved 34K gene. The 34K gene spans the intronic region of the ORF6/7 gene. Next to the 34K gene, four novel putative genes (ORF-A to -D) were predicted, as for canine adenoviruses, BtAdV-2 and BtAdV-3, although the functions of the putative protein products are unknown [25].

### 3.4. Sequence and Phylogenetic Analysis

**Figure 3:**
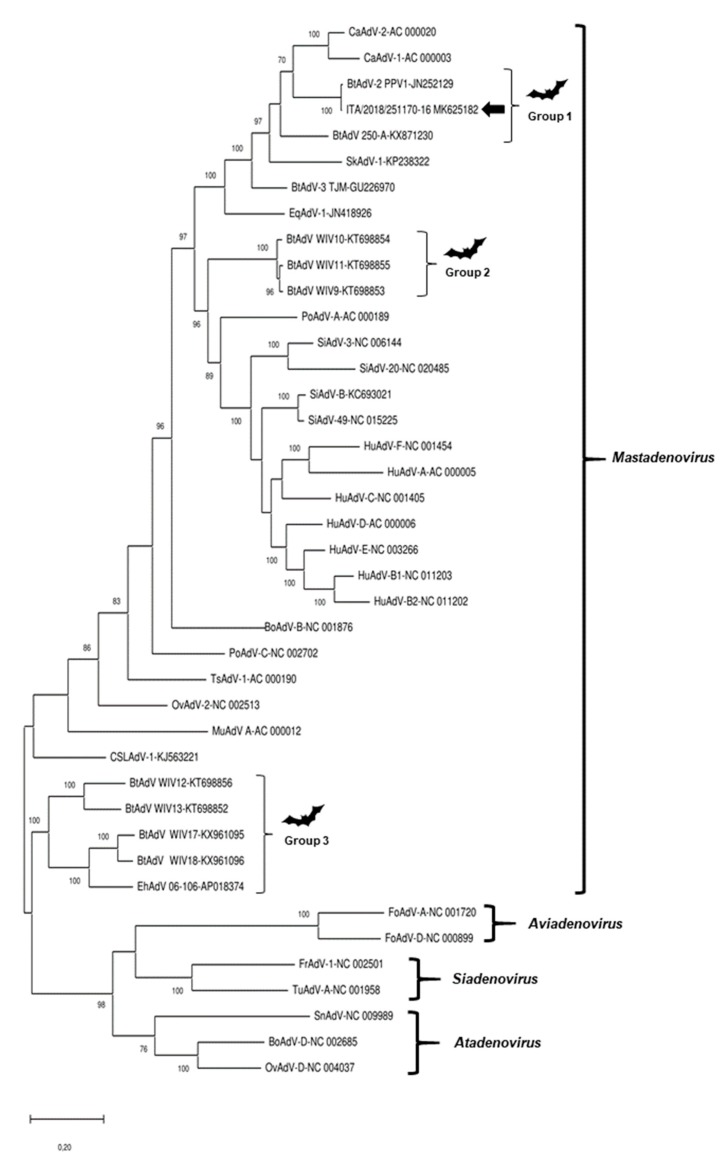
Phylogenetic tree based on the full-length genome of representative members of *Mastadenovirus*, *Aviadenovirus*, *Siadenovirus*, and *Atadenovirus* genera. GenBank accession numbers are provided for reference strains. The tree was generated using the maximum-likelihood method with the Jukes–Cantor algorithm of distance correction, with bootstrapping up to 1000 replicates. Bootstrap values >70% are shown. Scale bar indicates nt substitutions per site. Black arrow indicates the strain retrieved in this study. CaAdV, canine adenovirus, BtAdV, bat adenovirus, SkAdV, skunk adenovirus, EqAdV, equine adenovirus, PoAdV, porcine adenovirus, BoAdV, bovine adenovirus, SiAdV, simian adenovirus, HuAdV, human adenovirus, TsAdV, tree shrew adenovirus, OvAdV, ovine adenovirus, MuAdV, murine adenovirus, CSLAdV, California sea lion adenovirus, FoAdV, fowl adenovirus, FrAdV, frog adenovirus, TuAdV, turkey adenovirus, SnAdV, snake adenovirus.

Genomic analysis of the full-genome of the isolate ITA/2018/251170-16 revealed a 99.3% nucleotide (nt) identity to the reference strain PPV1. Upon phylogenetic analysis with 40 cognate full-genome sequences, the virus was tightly clustered with strain PPV1 (GenBank accession no. JN252129) and it was grouped with other bat strains, 250-A (GenBank accession no. KX871230) and TJM (GenBank accession no. GU226970), and with the canine adenoviruses (Figure 3).

**Figure 4:**
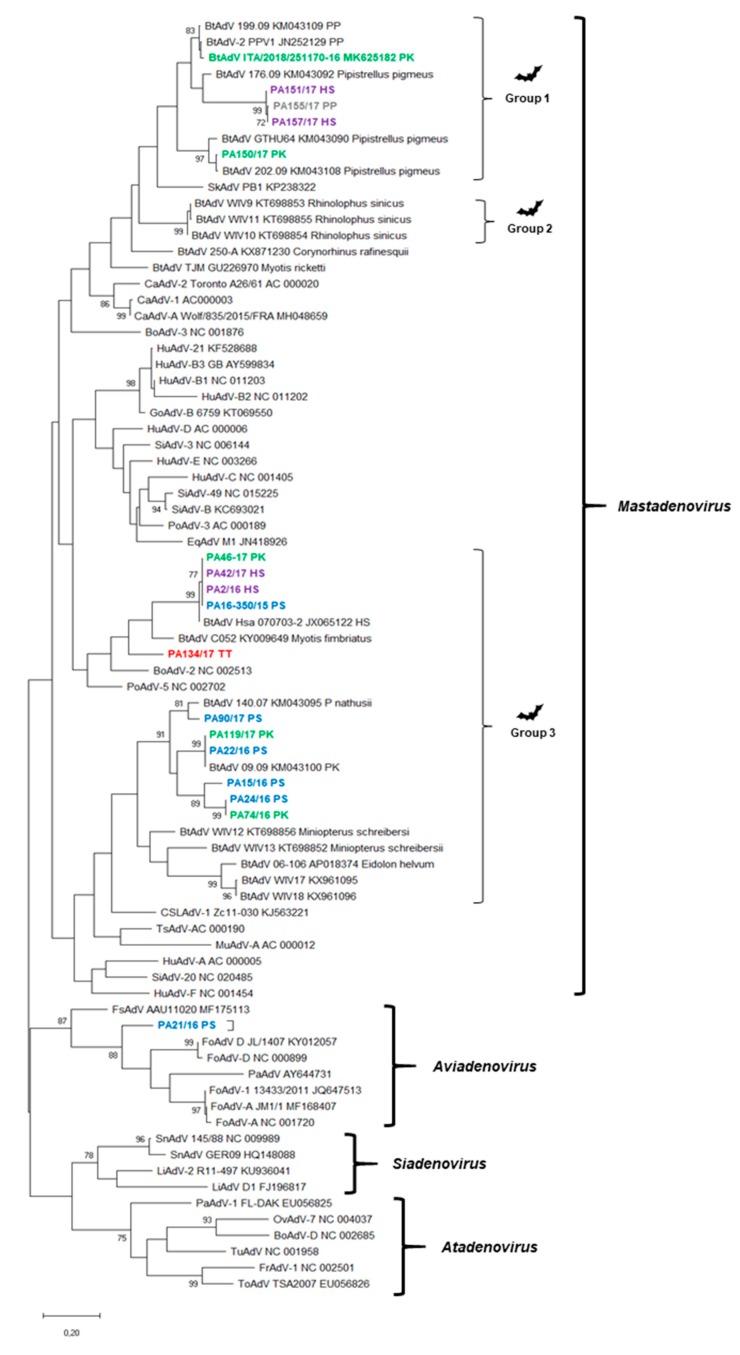
Phylogenetic tree based on the partial polymerase-coding region (233 nt positions) of adenoviruses. GenBank accession numbers are provided for reference strains. The tree was generated using the maximum-likelihood method with the Jukes–Cantor method by bootstrapping over 1000 replicates. Bootstrap values >70% are shown. Scale bar indicates nt substitutions per site. Blackarrows indicates the strains sequenced in this study. PS, *Pipistrellus* spp. (blue color); PP, *Pipistrellus pipistrellus* (grey color); PK, *Pipistrellus kuhlii* (green color); HS, *Hypsugo savii* (purple color); TT, *Tadarida teniotis* (red color). BtAdV, bat adenovirus, SkAdV, skunk adenovirus, EqAdV, equine adenovirus, CaAdV, canine adenovirus, HuAdV, human adenovirus, GoAdV, gorilla adenovirus, SiAdV, simian adenovirus, PoAdV, porcine adenovirus, MuAdV, murine adenovirus, TsAdV, tree shrew adenovirus, CSLAdV, California sea lion adenovirus, BoAdV, bovine adenovirus, FsAdV, fur seal adenovirus, PaAdV, parrot adenovirus, FoAdV, fowl adenovirus, SnAdV, snake adenovirus, LiAdV, lizard adenovirus, OvAdV, ovine adenovirus, TuAdV, turkey adenovirus, ToAdV, tortoise adenovirus, FrAdV, frog adenovirus.

Among the samples testing positive for adenoviral DNA by diagnostic PCR, 16 samples generated sequences of a good quality. The sequences were compared with cognate sequences available in the databases using FASTA interrogation [35] and the results are presented on Table 2. Genome sequences of the polymerase-coding region from 62 representative adenovirus strains were retrieved from GenBank and phylogenetic analysis was performed after sequence alignment (Figure 4). In this analysis, a unique bat adenovirus strain (PA21/16 PS) rooted along with a novel South American fur seal (*Arctocephalus australis*) adenovirus (GenBank accession no. MF175113), with which it shared 72.9% nt identity. Both the bat strain PA21/16 PS and the fur seal adenovirus were clustered tightly with bird adenoviruses (genus *Aviadenovirus*), sharing 67.6% to 72.1% nt identity in the small polymerase region sequence.

## 4. Discussion

In recent years, several novel viruses have been identified in bats [36,37]. Due to their potential role as carriers of zoonotic viral agents, public health agencies have intensified research studies on the bat virome with both pilot studies and large structured epidemiological investigations. In this study, we report the detection and analysis of BtAdV species in Northern Italy. The phylogenetic relationships with known BtAdVs were assessed, based on a short diagnostic fragment of the highly-conserved DNA polymerase gene. A BtAdV isolate was obtained from the carcass of *Pipistrellus kuhlii* presented with symptoms of inappetence and sensory depression, found in the province of Bergamo, Italy and dead after hospitalization in a wildlife rescue center.

Overall, we screened eight species of bats for the presence of adenoviral DNA, obtaining positive results in five species from three distinct bat genera. The overall adenovirus prevalence was 17.4% in the examined bats. A limit of our investigation was the heterogeneous composition of the sample collection, chiefly in terms of bat species, as the major part of the samples (186/195, 95.4%) derived from the genera *Pipistrellus* (136/195, 69.7%) and *Hypsugo* (50/195, 25.6%) and only 9/195 (4.6%) originated from the genera *Plecotus* (3/195, 1.5%), *Tadarida* (1/195, 0.5%), and *Eptesicus* (5/195, 2.6%). Nevertheless, the sample’s metadata were informative enough to suggest a possible age-related pattern of the infection, suggesting that adult animals are more prone to infection (*p* < 0.05, χ^2^ test).

Phylogenetic investigation of 17 strains sequenced in this study revealed a high diversity for adenoviruses present in bats, similar to previous studies in Europe, Asia, and Africa [20,23,24,37]. The impressive genetic variety of adenoviruses in bats poses a challenge for their nomenclature and classification. Upon phylogenetic analysis of the short diagnostic region spanning the DNA polymerase gene, the 17 strains were divided into at least 9 clusters/sub-clusters. Interestingly, bats from the genera, *Pipistrellus* and *Hypsugo*, appeared to share similar adenoviruses, suggesting inter-specific bat-to-bat circulation of adenoviruses.

Three strains (PA151/17, PA155/17, and PA157/17) identified in *Hypsugo savii* and *Pipistrellus pipistrellus* displayed 98.5% to 99.5% nt identity to each other and shared <72.6% nt identity with the BtAdV-2 strain PPV1 (GenBank accession no. JN252129), forming a separate cluster and suggesting a potential novel BtAdV species. More interestingly, a BtAdv strain (PA21/16 PS), detected in *Pipistrellus spp*., rooted tightly with a unique adenovirus found in South American fur seals (SAFS) pups (*Arctocephalus australis*) in Perù (GenBank accession number MF175113), and with avian adenoviruses (genus *Aviadenovirus*). The SAFS adenovirus was identified during a surveillance study for adenoviruses in breeding colonies of SAFS and Humboldt penguins (*Spheniscus humboldti*) in Southern America. In the Southern American study, four mastadenoviruses, four aviadenoviruses and a siadenovirus were detected in SAFSs, whilst three mastadenoviruses, two aviadenoviruses, and three siadenoviruses were identified in Humboldt penguins that shared the same reproductive area in the Peruvian coast [38]. These findings are intriguing, as they indicate the possibility of inter-species transmission between avian and mammalian species and in our study this could have been accounted for by the fact that bats and birds may reach/occupy the same ecological niches (i.e., terminal branches of trees, rocks, or caves) due to their flying abilities. The possibility of a shift between avian and mammalian hosts for adenoviruses is not considered a common event, since adenoviruses are considered highly species-specific [3,7]. Yet, members of the *Atadenovirus* genus have been identified in distantly related animal species, including ruminants, poultry, reptiles, and a marsupial [39]. An additional hypothesis is that avian adenovirus DNA may contaminate bat food and that the detected virus was not replicating actively in the animal. Regardless, the resolution of the DNA polymerase region fragment targeted by the consensus PCR is likely not sufficient to discriminate firmly among the adenovirus genera and species and this preliminary piece of evidence should be confirmed by gathering larger, more informative sequences or, possibly, the full-length genome.

An isolate, strain ITA/2018/251170-16, was obtained on simian cell lines and its full-length sequence was generated using deep sequencing technology. When reconstructing the sequence and genome organization, the virus appeared virtually identical to the prototype strain PPV1 (GenBank accession no. JN252129), sharing 99.3% nt identity. Such a high nt identity between those two viruses is intriguing, as the two viruses have been identified approximately a decade apart from each other and in different geographical European countries, respectively, Germany and Italy [24]. Similar degrees of sequence conservation over time, geographical locations, and host species have been documented for canine adenoviruses type 1 and type 2. Both the canine viruses have long been recognized as canine pathogens and they are also able to infect several wild carnivores, with minimal genetic differences among the various isolates, suggesting a limited genetic diversification, likely due to optimal adaptation of those viruses to carnivores [27,40].

In conclusion, by screening samples from bats in Northern Italy, we observed that adenoviruses are common agents of the bat virome. Also, an impressive genetic diversity was observed for bat adenoviruses, despite the relatively small population of bats included in our survey. The use of consensus, broadly reactive, primer sets for the *Adenoviridae* family allowed the detection of an avian-like virus, an apparent exception to the adenovirus literature, already observed elsewhere [38] but still requiring confirmatory data.

## Figures and Tables

**Table 1:** Classification (2018) of bat mastadenovirus species proposed by ICTV.

Strain	Species	GenBank Accession No.	Reference
TJM	*Bat mastadenovirus A*	GU226970	[25]
PPV1	*Bat mastadenovirus B*	JN252129	[24]
WIV9	*Bat mastadenovirus C*	KT698853	[21]
WIV12	*Bat mastadenovirus D*	KT698856	[2]
WIV13	*Bat mastadenovirus E*	KT698852	[2]
WIV17	*Bat mastadenovirus F*	KX961095	[2]
250-A	*Bat mastadenovirus G*	KX871230	[22]
EhAdV 06-106	Bat mastadenovirus H *	JX885602	[23]

* Candidate novel species.

**Table 2:** BLAST investigation results for the 16 partial polymerase-coding region sequences generated. All the sequences were generated from samples consisted of pools of organs, except for strain PA16-350/15 PS that originated from a fecal sample. Gray shades indicate similarities among strains. The nt identity to the closest adenovirus strains is also indicated.

		Results of FASTA Interrogation				Pairwise Identity among the Italian Viruses	
Strain (Id lab)	Bat species	Identity/*p*-value	GenBank accession no.	Name/species	Country	Identity	Strain
PA2/16 HS (189/18-2)	*Hypsugo savii*	99.5%/1.7E-106	JX065122	Hsa_070703-2/*Hypsugo savii*	Spain (2007)	99.5%	PA42, PA46, PA16-350/15
PA42/17 HS (189/18-42)	*Hypsugo savii*	100.0%/8.2E-106	JX065122	Hsa_070703-2/*Hypsugo savii*	Spain (2007)	99.5–100.0%	PA2, PA46, PA16-350/15
PA46/17 PK (189/18-46)	*Pipistrellus kuhlii*	100.0%/7.1E-106	JX065122	Hsa_070703-2/*Hypsugo savii*	Spain (2007)	99.5–100.0%	PA2, PA42, PA16-350/15
PA16-350/15 PS (350/15-16)	*Pipistrellus* spp.	99.5%/1.5E-107	JX065122	Hsa_070703-2/*Hypsugo savii*	Spain (2007)	99.5%	PA2, PA42, PA46
PA15/16 PS (189/18-15)	*Pipistrellus* spp.	76.1%/1.1E-45	KM043095	140/07/*Pipistrellus nathusii*	Germany (2007)	86.2–86.7%	PA24, PA74
PA24/16 PS (189/18-24)	*Pipistrellus* spp.	76.1%/1.1E-40	KM043095	140/07/*Pipistrellus nathusii*	Germany (2007)	99.5%	PA74
PA74/16 PK (189/18-74)	*Pipistrellus kuhlii*	76.1%/5.1E-42	KM043095	140/07/*Pipistrellus nathusii*	Germany (2007)	99.5%	PA24
PA21/16 PS (189/18-21)	*Pipistrellus* spp.	74.0%/4.2E-51	MF175113	AAU11020/*Arctocephalus australis*	Peru (2009)	52.1–52.5%	PA150, PA151
PA22/16 PS (189/18-22)	*Pipistrellus* spp.	100.0%/4.7E-82	KM043100	09/09/*Pipistrellus kuhlii*	Germany (2009)	100.0%	PA119
PA119/17 PK (189/18-119)	*Pipistrellus kuhlii*	100.0%/1.2E-81	KM043100	09/09/*Pipistrellus kuhlii*	Germany (2009)	100.0%	PA22
PA90/17 PS (189/18-90)	*Pipistrellus* spp.	91.3%/1.3E-72	KM043095	140/07/*Pipistrellus nathusii*	Germany (2007)	77.6%	PA22, PA119
PA134/17 TT (189/18-134)	*Tadarida teniotis*	77.6%/5.0E-47	KY009649	C052/*Myotis fimbriatus*	China (2015)	70.3%	PA150
PA150/17 PK (189/18-150)	*Pipistrellus kuhlii*	99.1%/7.5E-102	KM043108	202/09/*Pipistrellus pipistrellus*	Germany (2009)	72.1–72.6%	PA2, PA42, PA46, PA151, PA16-350/15
PA151/17 HS (189/18-151)	*Hypsugo savii*	76.0%/5.8E-54	KM043092	176/09/*Pipistrellus pygmaeus*	Germany (2009)	98.6–99.1%	PA155, PA157
PA155/17 PP (189/18-155)	*Pipistrellus pipistrellus*	74.7%/5.0E-49	KM043092	176/09/*Pipistrellus pygmaeus*	Germany (2009)	98.6–99.5%	PA151, PA157
PA157/17 HS (189/18-157)	*Hysugo savii*	75.1%/6.7E-50	KM043092	176/09/*Pipistrellus pygmaeus*	Germany (2009)	99.1–99.5%	PA151, PA155
